# Understanding the factors governing the water oxidation reaction pathway of mononuclear and binuclear cobalt phthalocyanine catalysts†

**DOI:** 10.1039/d2sc02213c

**Published:** 2022-07-08

**Authors:** Qing'e Huang, Jun Chen, Peng Luan, Chunmei Ding, Can Li

**Affiliations:** Department of Chemical Physics, University of Science and Technology of China Hefei 230026 China canli@dicp.ac.cn; State Key Laboratory of Catalysis, Dalian Institute of Chemical Physics, Chinese Academy of Sciences Dalian 116023 China; University of Chinese Academy of Sciences Beijing 100049 China

## Abstract

The rational design of efficient catalysts for electrochemical water oxidation highly depends on the understanding of reaction pathways, which still remains a challenge. Herein, mononuclear and binuclear cobalt phthalocyanine (mono-CoPc and bi-CoPc) with a well-defined molecular structure are selected as model electrocatalysts to study the water oxidation mechanism. We found that bi-CoPc on a carbon support (bi-CoPc/carbon) shows an overpotential of 357 mV at 10 mA cm^−2^, much lower than that of mono-CoPc/carbon (>450 mV). Kinetic analysis reveals that the rate-determining step (RDS) of the oxygen evolution reaction (OER) over both electrocatalysts is a nucleophilic attack process involving a hydroxy anion (OH^−^). However, the substrate nucleophilically attacked by OH^−^ for bi-CoPc is the phthalocyanine cation-radical species (Co^II^–Pc–Pc˙^+^–Co^II^–OH) that is formed from the oxidation of the phthalocyanine ring, while cobalt oxidized species (Pc–Co^III^–OH) is involved in mono-CoPc as evidenced by the operando UV-vis spectroelectrochemistry technique. DFT calculations show that the reaction barrier for the nucleophilic attack of OH^−^ on Co^II^–Pc–Pc˙^+^–Co^II^–OH is 1.67 eV, lower than that of mono-CoPc with Pc–Co^III^–OH nucleophilically attacked by OH^−^ (1.78 eV). The good agreement between the experimental and theoretical results suggests that bi-CoPc can effectively stabilize the accumulated oxidative charges in the phthalocyanine ring, and is thus bestowed with a higher OER performance.

## Abstract 2

bi-CoPc can stabilize accumulated oxidative charges in phthalocyanine ring, which leads to the OER proceeding through a nucleophilic attack of OH^^-^^ on the phthalocyanine cation-radical species that is formed from the oxidation of phthalocyanine ring.

## Graphical abstract

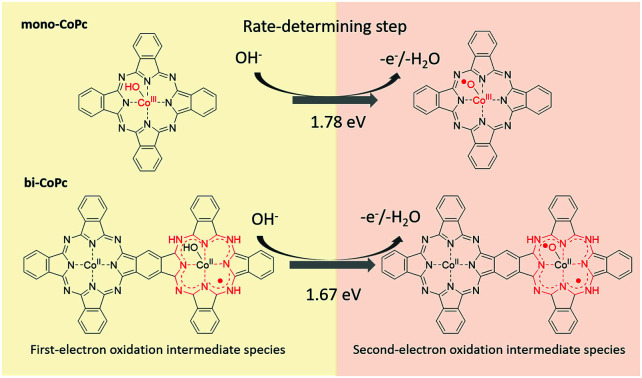

## Introduction

Hydrogen production *via* electrocatalytic water splitting utilizing renewable energy is recognized as one of the most intriguing routes to address energy and environmental problems.^1–3^ The key challenge and long-standing issue is to facilitate the oxygen evolution reaction (OER), which is thermodynamically unfavorable and kinetically sluggish, and largely limits the rate of water splitting.^4–6^ Although OER electrocatalysts have been widely explored for decades, the intrinsic catalytic mechanism is still unclear because of the complicated active sites and reaction pathways, especially for heterogeneous electrocatalysts.^7–12^

Taking advantage of the well-defined coordination structure, homogeneous metal complexes have been developed as typical OER catalysts and models for understanding the fundamental reaction pathways of water oxidation.^13–19^ For example, a copper bipyridine complex was reported for water oxidation, and Cu(iii)–OH intermediate species was determined to be responsible for O–O bond formation *via* a water nucleophilic attack mechanism.^20,21^ Besides, metal complexes with macrocyclic ligands, such as porphyrin and corrole ligands,^17,22,23^ were also investigated as OER catalysts owing to their robust coordination environment during the oxidation process, in which O–O bond formation proceeds mostly through a macrocyclic radical cation intermediate species and the macrocyclic ligand could serve as an oxidation charge reservoir for the OER.

Inspired by the structure of the CaMn_4_O_5_ oxygen evolution center in nature,^24,25^ it has been anticipated that binuclear and multinuclear complexes could catalyze water oxidation efficiently. A blue dimer with a μ-O bridge is the first example of a binuclear complex reported for the OER,^26^ which catalyzes O–O bond formation through water nucleophilic attack on a single Ru site to generate Ru(ii)–OOH, but nevertheless, the oxidation charges are stored in two metal sites (one-site catalysis with a two-site oxidation mechanism). To improve the stability of catalysts during the water oxidation process, an extended π conjugated bridge between two metal sites was introduced to prevent ligands from oxidizing, such as benzene and alkenyl bridged binuclear metal complexes.^27–29^ Importantly, with the help of the π conjugated bridge, these catalysts show one-site catalysis with a two-site oxidation mechanism. As described above, both the macrocyclic ligand and metal site could play the role of a reservoir of oxidation charge. Therefore, macrocyclic binuclear metal complexes bridged by a π conjugated ligand are equivalent to multinuclear catalysts, which are identified as potential efficient OER catalysts.^30^ However, the reaction pathway and the structure–activity relationship of these binuclear metal macrocyclic complexes are still not well understood, and they need to be investigated and deciphered in detail.

In this study, using mono-CoPc and bi-CoPc, we investigated the role of the electronic structure in the OER pathway. It is shown that bi-CoPc catalyzes water oxidation more efficiently compared with mono-CoPc as demonstrated by the electrocatalytic and photocatalytic OER tests. A catalytic mechanism involving a rate-determining step (RDS) with the first-electron oxidation intermediate species nucleophilically attacked by OH^−^ is proposed for both catalysts based on the reaction kinetic analysis. The intermediate species that triggers the RDS for bi-CoPc is the phthalocyanine ring oxidized species (Co^II^–Pc–Pc˙^+^–Co^II^–OH), while Pc–Co^III^–OH species with an oxidized cobalt center is involved in the RDS for mono-CoPc. The higher catalytic OER performance of bi-CoPc is attributed to its high capacity for stabilizing the accumulated oxidation charges in the phthalocyanine ring, thus affording a phthalocyanine cation radical (Co^II^–Pc–Pc˙^+^–Co^II^–OH) as intermediate species and a lower reaction barrier pathway for bi-CoPc.

## Results and discussion

### Electronic structure characterization of mono-CoPc and bi-CoPc

**Fig. 1:**
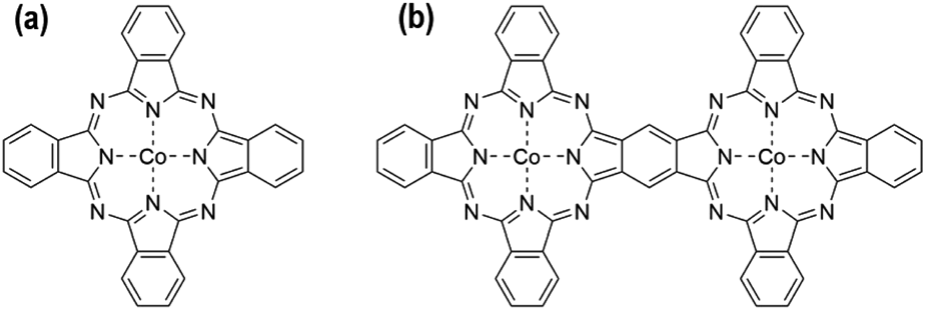
The molecular structure of (a) mono-CoPc and (b) bi-CoPc.

The detailed synthesis process of bi-CoPc/carbon and mono-CoPc/carbon is described in the Experimental section. Fig. 1a and b show the molecular structures of mono-CoPc and bi-CoPc. Specifically, the binuclear metal sites are 8 atoms apart from each other in bi-CoPc. The structures of both molecules were verified clearly by UV-vis^31^ and FT-IR spectra^32,33^ (Fig. S1a and b†). Importantly, the higher delocalization of bi-CoPc is shown in the UV-vis spectra (Fig. S1a†), where bi-CoPc exhibits a new band at 693 nm that can be assigned to electronic interaction and delocalization between the halves of the phthalocyanine units.^33–35^ The local structure of Co atoms in mono-CoPc and bi-CoPc was further characterized by the X-ray absorption technique. The Fourier-transformed curves of the extended X-ray absorption fine structure (EXAFS) spectra for mono-CoPc and bi-CoPc demonstrate that the Co atomic environment in both mono-CoPc and bi-CoPc consists of N in the first coordination shells with average coordination numbers of 4.2 ± 0.2 and 4.1 ± 0.2, respectively, consistent with the molecular structures in Fig. 1 (Fig. S2 and Table S1†).

Scanning electron microscopy (SEM) was used to characterize the morphology of mono-CoPc/carbon and bi-CoPc/carbon. Fig. S3a and b† show that mono-CoPc and bi-CoPc molecules have an aggregate stratified structure, and the carbon support has a nanoparticle structure in the range of 50–100 nm (Fig. S4a†). Mono-CoPc/carbon and bi-CoPc/carbon exhibit a structure similar to that of the carbon support (Fig. S4b and c†), which is totally different from the stacked structure of mono-CoPc and bi-CoPc, signifying that the aggregation of mono-CoPc and bi-CoPc molecules is prevented by the existence of a carbon support. This is in line with the result of X-ray power diffraction (XRD) that only carbon support signals are observed for mono-CoPc/carbon and bi-CoPc/carbon (Fig. S5†).

**Fig. 2:**
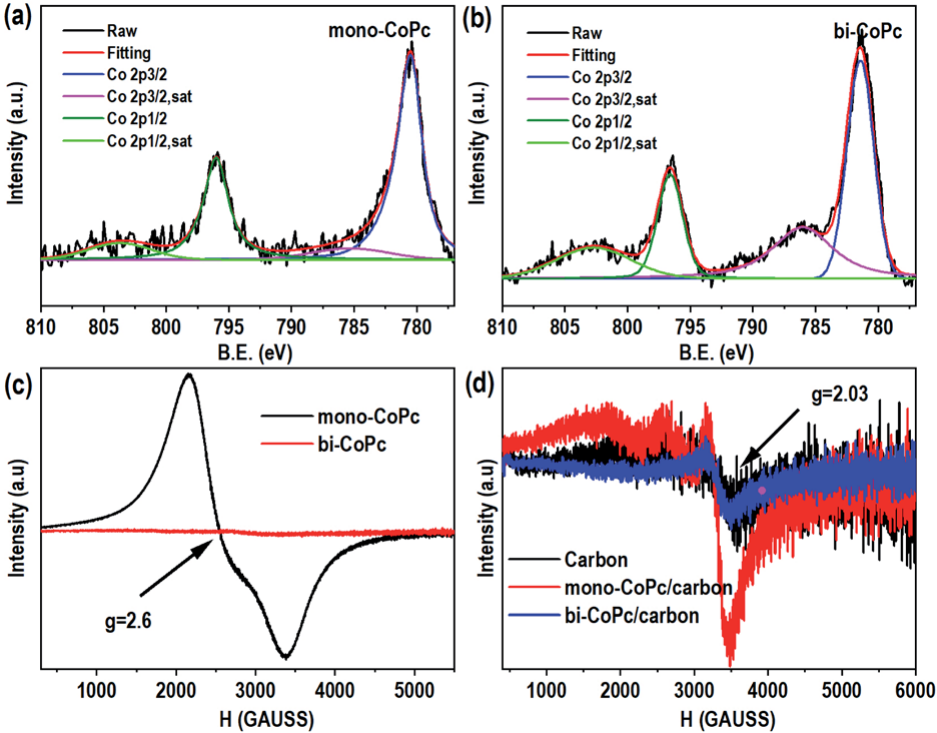
The structural characterization of catalysts. The Co 2p XPS spectra for (a) mono-CoPc and (b) bi-CoPc. EPR spectra (77 K) of catalysts without a carbon support (c) and (d) with a carbon support.

Furthermore, X-ray photoelectron spectroscopy (XPS) was conducted to explore the elementary composition and electronic structure of these catalysts (Fig. 2a, b, S6 and S7†). Mono-CoPc shows two peaks of Co 2p_3/2_ at 780.5 eV and Co 2p_1/2_ at 796.0 eV with an Δ*E* value of 15.5 eV, and an energy interval of 4.82 eV between Co 2p_3/2_ and the corresponding satellite peak is observed, demonstrating that the Co species in mono-CoPc is mainly Co^2+^ (Fig. 2a).^36,37^ For bi-CoPc, the Co species is also confirmed to be Co^2+^ as the binding energy and XPS profile only show some slight differences compared with mono-CoPc (Fig. 2b). And the valence state of Co in these two catalysts does not change after immobilizing mono-CoPc or bi-CoPc onto a carbon support (Fig. S6a and b†).

To further investigate the chemical valence state and electron spin state of Co in mono-CoPc/carbon and bi-CoPc/carbon complexes, electron paramagnetic resonance (EPR) with high sensitivity to coordination environments was used to acquire information on unpaired electrons. Fig. 2c shows only one signal (*g* = 2.6) indexed to the low spin Co^2+^ component in mono-CoPc,^36,38,39^ which coincides well with the XPS results. As for bi-CoPc, no apparent EPR signal is observed when compared with mono-CoPc, usually illustrating the existence of Co^3+^ components.^40^ However, an alternative explanation for the absence of an EPR signal is the phenomenon of racemization, which appears under the condition that two metal atoms with unpaired electrons are located very close.^41^ In this case, the existence of electronic interaction and delocalization between the halves of the Pc units in bi-CoPc makes the Co atoms interact with each other, and thus we think that bi-CoPc exhibited a racemization phenomenon.^41,42^ Combining the results of XPS, we conclude that the chemical valence state of Co species in bi-CoPc are also +2. When immobilizing mono-CoPc or bi-CoPc molecules onto a carbon support, the hyperfine splitting phenomenon of Co^2+^ is manifested in mono-CoPc/carbon (Fig. 2d), which is ascribed to the prevention of aggregation of mono-CoPc complexes by the presence of a carbon support. For bi-CoPc/carbon, it shows a similar EPR signal with a carbon support, signifying that the introduction of a carbon support did not alter the Co species in bi-CoPc.

**Fig. 3:**
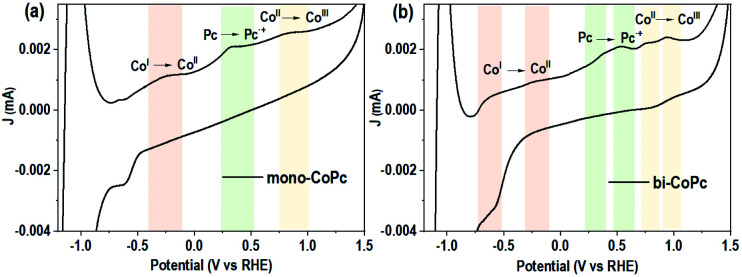
Circulation voltammetry (CV) curves of (a) mono-CoPc and (b) bi-CoPc in MeCN solution, using 0.1 M TBAPF_6_ as the supporting electrolyte and glassy carbon as a working electrode.

As mentioned above, the electronic structures of mono-CoPc and bi-CoPc are different, which might confer a different redox behavior to these catalysts. The redox properties of mono-CoPc and bi-CoPc were investigated by means of cyclic voltammetry (CV) in MeCN (Fig. 3). Careful inspection indicates that three oxidation couples at −0.264, 0.322 and 0.822 V *vs.* ferrocene (Fc) are involved for mono-CoPc (Fig. 3a),^43^ and the splitting of all three oxidation couples in bi-CoPc are observed as well. The oxidation processes of Co(ii)/Co(i) at −0.264 V and Co(iii)/Co(ii) at 0.822 V *vs.* Fc for mono-CoPc lie midway between the split waves of the binuclear oxidation couple, while the oxidation process of the Pc ring at 0.322 V *vs.* Fc for mono-CoPc appears at a more negative potential than the corresponding oxidation peaks in bi-CoPc (0.385 and 0.583 V *vs.* Fc). The lower oxidation potential of the ligand means that the ligand is easily oxidized and thus confers lower structural stability to the metal complex. Therefore, the location of Pc ring oxidation for mono-CoPc and bi-CoPc shows that the Pc ring in bi-CoPc is more stable than that in mono-CoPc, which is attributed to the extended delocalization of the binuclear structure.

### OER catalytic performance

**Fig. 4:**
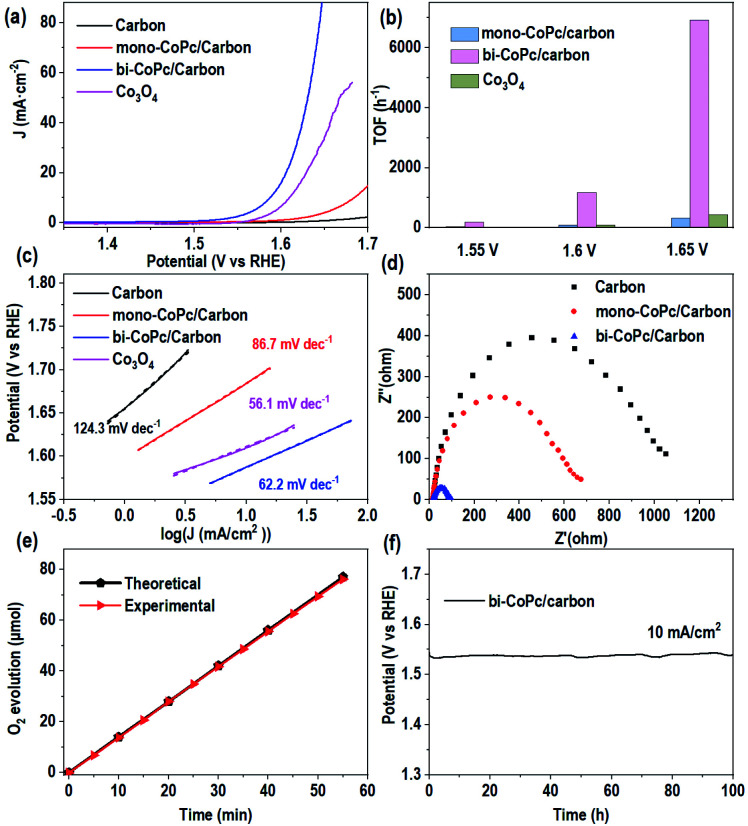
Electrochemical performance of catalysts in 1 M KOH electrolyte. (a) Linear sweep voltammetry (LSV) curves of catalysts at a scan rate of 5 mV s^−1^. (b) Turnover frequency (TOF) values of catalysts at different potentials (*vs.* RHE). (c) Tafel plots of catalysts. (d) Nyquist plots of mono-CoPc and bi-CoPc measured at a potential of 1.6 V *vs.* RHE. (e) The faradaic efficiency of bi-CoPc/carbon, which can be calculated from the theoretical and experimental molar number of O_2_ produced. (f) The chronopotentiometry curves of bi-CoPc/carbon at an anodic current density of 10 mA cm^−2^.

Based on the above results and analysis, we tried to unravel the structure–activity relationship of both catalysts in the OER. The electrocatalytic performances of the catalysts were evaluated by using a three-electrode system in 1 M KOH. Fig. 4a shows that the carbon support shows a poor OER performance and the introduction of mono-CoPc onto the carbon support can improve the OER activity slightly, but an overpotential of more than 450 mV at 10 mA cm^−2^ is required for these two catalysts. However, bi-CoPc/carbon shows a much lower overpotential of 357 mV at 10 mA cm^−2^. In addition, we also evaluated the water oxidation performance of Co_3_O_4_ with a much higher loading amount of Co sites and revealed that bi-CoPc/carbon can catalyze water oxidation more efficiently than Co_3_O_4_. For comparison, the intrinsic performance is calculated for all catalysts, and the turnover frequency (TOF) values at 1.6 V for mono-CoPc/carbon, bi-CoPc/carbon and Co_3_O_4_ are 84, 1170, and 86 h^−1^, respectively (Fig. 4b and S8†), further demonstrating that bi-CoPc/carbon is the most efficient catalyst for the OER. Fig. 4c shows the Tafel slope values of 124, 86.7 and 62.2 mV dec^−1^ for the carbon support, mono-CoPc/carbon and bi-CoPc/carbon, respectively, indicating that bi-CoPc/carbon has the fastest reaction kinetics for the OER. Moreover, Nyquist plots were used to explore the interfacial charge transfer behavior of these catalysts (Fig. 4d). The arc radius corresponding to the interfacial charge transfer resistance (*R*_ct_) for bi-CoPc/carbon is smaller than that of other components, meaning a faster electrode–electrolyte interface charge transfer for bi-CoPc/carbon. The reason for the smaller charge transfer resistance in bi-CoPc/carbon is probably that bi-CoPc has a higher electrical conductivity than mono-CoPc, which results from the extended π-delocalized macrocyclic ligand in the binuclear catalyst. A faradaic efficiency of ∼100% up till 1.68 V (*vs.* RHE) for bi-CoPc/carbon signifies that only the water oxidation reaction proceeds during the electrocatalytic reaction (Fig. 4e and S9†). The stability of mono-CoPc and bi-CoPc/carbon was also evaluated, indicating that bi-CoPc/carbon is stable at least for 100 h under reaction conditions and the degradation or detachment of this catalyst did not happen in a long-term reaction (Fig. 4f and S10†). The structure rigidity of the catalyst and the absence of CoO_*x*_ at the surface of the bi-CoPc/carbon electrode during the electrocatalytic reaction are evidenced by the same Co 2p spectra of bi-CoPc/carbon before and after electrocatalytic measurement (Fig. S11†). This is consistent with the results of X-ray power diffraction (XRD) and HRSEM that no new species are observed for bi-CoPc/carbon after OER measurement (Fig. S12 and S13†). In addition, the absence of a peak corresponding to metal oxide in the O 1s spectrum for bi-CoPc/carbon and mono-CoPc/carbon after OER measurement also verifies their stability during the LSV test (Fig. S14†).^44^

Meanwhile, the photocatalytic water oxidation activities of mono-CoPc and bi-CoPc were also evaluated in the Ru(bpy)_3_^2+^–Na_2_S_2_O_8_ system,^45^ and bi-CoPc exhibits a much higher oxygen evolution rate compared with mono-CoPc (Fig. S15†). These results show that the electronic structure of active sites in mono-CoPc/carbon and bi-CoPc/carbon is decisive in regulating the OER activity, which needs to be carefully explored and deciphered in future research.

### Study of the water oxidation mechanism of mono-CoPc and bi-CoPc

**Fig. 5:**
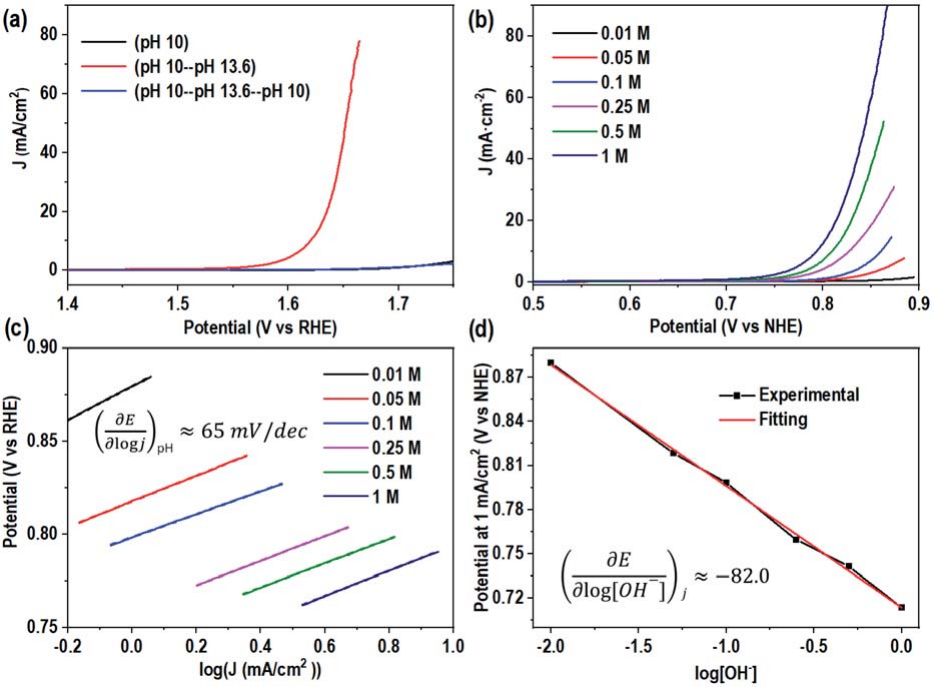
Electrokinetic study of bi-CoPc/carbon. (a) The reversible catalytic OER performance with a change in the KOH concentration. This experiment is conducted as follows: Step 1: evaluating the electrocatalytic OER activity of bi-CoPc/carbon in an electrolyte with pH 10; Step 2: then, the bi-CoPc/carbon electrode was transferred to another electrolyte with pH 13.6 for testing; Step 3: finally, this electrode was transferred back to the electrolyte with pH 10 for the OER test. (b) Electrocatalytic performance of bi-CoPc/carbon in KOH electrolytes with different concentrations. The pH values for 1.00, 0.50, 0.25, 0.10, 0.05, and 0.01 M KOH electrolytes are 13.9, 13.7, 13.4, 13.1, 12.8, and 12.1, respectively. (c) Tafel plots of KOH electrolytes with different concentrations. (d) Fitting plots of different applied potentials at 1 mA cm^−2^*versus* the logarithm of [OH^−^].

The interesting difference in water oxidation performance between mono-CoPc/carbon and bi-CoPc/carbon motivates us to further study the mechanism of both catalysts in the water oxidation process. To explore the RDS of water oxidation on bi-CoPc/carbon, an electrokinetic study of catalysts in various concentrations of KOH (0.01 to 1 M) was carried out. Here, the structural stability of bi-CoPc/carbon during electrocatalytic water oxidation is further confirmed by the reversible catalytic performance with the change in KOH concentrations (Fig. 5a). A Tafel slope of 65 mV dec^−1^ corresponding to a typical value of a cobalt-based catalyst for the OER in all concentrations of KOH is presented (Fig. 5b and c and Table S2†), suggesting that the RDS of water oxidation is a 1H^+^/1e^−^ proton coupling electron transfer (PCET) step.^23^ The OER activity shows a monotonic increase with increasing OH^−^ concentration (Fig. 5b), and the potential (*vs.* RHE) of 1 mA cm^−2^ decreases with the increase in the log[OH^−^] value with a slope of ∼80 mV (Fig. 5d). The current density has an approximately first-order behavior dependence on the concentration of OH^−^, indicating that only one OH^−^ is directly involved in the RDS.^46^ Meanwhile, we found that mono-CoPc/carbon exhibits the same dependency relationship with OH^−^ though an electrokinetic study (Fig. S16†).1
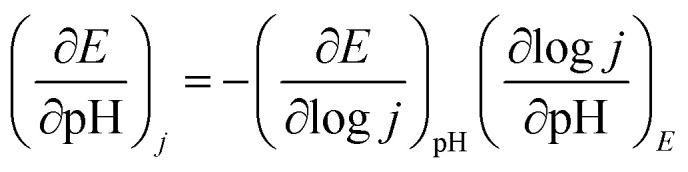


**Fig. 6:**
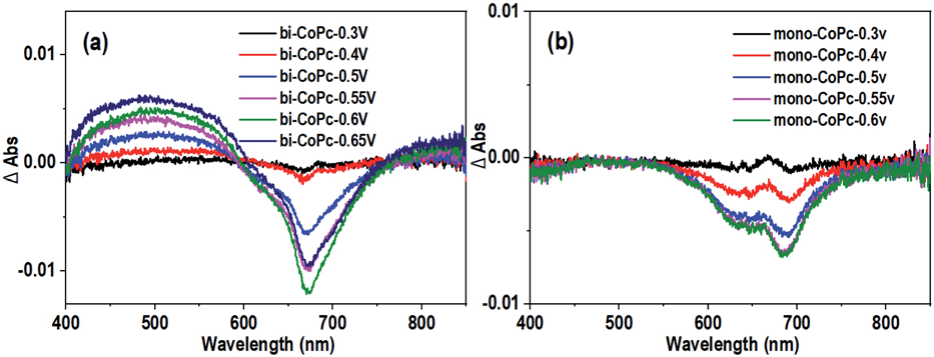
Differential spectra of UV–vis spectroelectrochemistry at different applied potentials *vs.* Ag/AgCl for (a) bi-CoPc and (b) mono-CoPc in 1 M KOH.

Furthermore, the UV–vis spectroelectrochemistry technique was utilized to tentatively explore the intermediate species in the water oxidation process. The initial bi-CoPc electrode exhibits two Q bands at 671 and 613 nm in the 1 M KOH electrolyte without an applied voltage (Fig. S17a†), and the intensity of these two bands decreases upon exerting increasing electrochemical oxidation potential on the electrode, indicating the consumption of the initial species. Meanwhile, the appearance of new broad absorbance bands in the region of 400–600 nm suggests the formation of new species (Fig. 6a), which require further confirmation by carefully analyzing the UV-vis spectra. During the oxidation of bi-CoPc, the emergence of a new broad absorbance band between the Soret and Q band (340–570 nm) has been previously considered as an indication of phthalocyanine ring oxidation,^47,48^ which corresponds to the phthalocyanine cation-radical species. Based on the foregoing analysis, the new intermediate species involved in the water oxidation RDS for bi-CoPc is considered to be cobalt phthalocyanine cation-radical species, denoted as Co^II^–Pc–Pc˙^+^–Co^II^–OH. With respect to the UV-vis spectrum of mono-CoPc, the absence of a characteristic peak associated with phthalocyanine cation–radical species indicates that this process is best described as Co^II^/Co^III^ (Fig. 6b and S17b†).

### Computational study

**Fig. 7:**
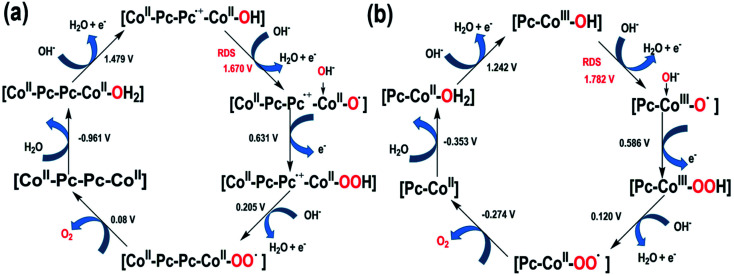
Proposed water oxidation mechanism for (a) bi-CoPc and (b) mono-CoPc.

We further performed DFT calculations to decipher the mechanism of water oxidation on mono-CoPc and bi-CoPc and the reason for the higher catalytic OER activity of bi-CoPc. A scheme of the water oxidation mechanism over mono-CoPc and bi-CoPc is proposed, as illustrated in Fig. 7.

As already discussed in the electrokinetic study, the RDS of water oxidation on bi-CoPc is a 1H^+^/1e^−^ PCET step and only one OH^−^ is involved in this step. DFT calculations demonstrate that the RDS proceeds through a nucleophilic attack of OH^−^ on Co^II^–Pc–Pc˙^+^–Co^II^–OH with a thermodynamic barrier of 1.67 eV (Fig. 7a, S18 and Table S3†), in good agreement with the experimental observation in the electrokinetic study. The involvement of the phthalocyanine cation-radical Co^II^–Pc–Pc˙^+^–Co^II^–OH species in the RDS is again consistent with our experimental results observed in UV-vis spectroelectrochemistry. As for mono-CoPc, the RDS is triggered by Pc–Co^III^–OH *via* a nucleophilic attack process of OH^−^ with a thermodynamic barrier of 1.78 eV (Fig. 7b, S19 and Table S4†), which is supported by the results of the electrokinetic study and UV-vis spectroelectrochemistry. As described above, bi-CoPc provides a lower reaction barrier for the OER (1.67 eV) compared with mono-CoPc (1.78 eV), which explains our experimental result of OER performance well.

The difference in the OER pathway for these two catalysts is the storage location of oxidation charge. The metal site acts as a reservoir of oxidation charge in mono-CoPc, and the phthalocyanine ring plays the role of a reservoir for oxidation charge in bi-CoPc. The distinctive reaction pathway for bi-CoPc might be attributed to the larger π electron cloud in bi-CoPc compared with that in mono-CoPc, which helps to stabilize the accumulated oxidative charges in the phthalocyanine ring, in good agreement with the experimental observation of redox behavior in CV measurement.

## Conclusions

In conclusion, taking mono-CoPc and bi-CoPc as examples, we found that bi-CoPc can catalyze water oxidation more efficiently than mono-CoPc. Based on the exploration of the electronic structure and the spectroelectrochemistry behavior of bi-CoPc and mono-CoPc in the OER, we proposed a reasonable catalytic mechanism for bi-CoPc and showed that the higher catalytic performance of bi-CoPc is attributed to the stronger capacity for stabilizing the accumulated oxidative charges in the phthalocyanine ring, and thus making the OER proceed through a nucleophilic attack of OH^−^ on Co^II^–Pc–Pc˙^+^–Co^II^–OH with a thermodynamic barrier of 1.67 eV. However, water oxidation on mono-CoPc proceeds through a nucleophilic attack of OH^−^ on Pc–Co^III^–OH with a high reaction barrier of 1.78 eV. This work discloses the effects of the electronic structure of active sites on the OER route and performance, which may guide the rational design of highly active catalysts.

## Data availability

All experimental and characterization data are available in the ESI.†

## Author contributions

Qing'e Huang and Can Li conceived the project. Qing'e Huang performed the experimental work and wrote the manuscript. Jun Chen performed the DFT calculations. All of the authors discussed the results and contributed to the preparation of the manuscript.

## Conflicts of interest

There are no conflicts to declare.

## Supplementary Material

SC-013-D2SC02213C-s001
