# Supplementary material for: Understanding the factors governing the water oxidation reaction pathway of mononuclear and binuclear cobalt phthalocyanine catalysts
Source: Chem Sci. 2022 Jul 8;13(30):8797–803. doi: 10.1039/d2sc02213c (PMC9350663; doi:10.1039/d2sc02213c)
Supplement: SC-013-D2SC02213C-s001 [file SC-013-D2SC02213C-s001.pdf]

Electronic Supplementary Material (ESI) for Chemical Science.
This journal is © The Royal Society of Chemistry 2022

Understanding the factors governing the water oxidation reaction pathway on mononuclear and binuclear cobalt phthalocyanine catalysts

Qing'e Huang [a, b], Jun Chen [b, c], Peng Luan [b], Chunmei Ding [b] and Can Li [a, b, c] *

[a] Department of Chemical Physics, University of Science and Technology of China, Hefei 230026, China

[b] State Key Laboratory of Catalysis, Dalian Institute of Chemical Physics, Chinese Academy of Sciences, Dalian 116023, China

[c] University of Chinese Academy of Sciences, Beijing 100049, China

Table of Contents

1. **Experimental Section ……………………………………………………………S3-S8**
2. **Catalyst characterizations (UV-vis, FT-IR, XAS and SEM)……………………………S9-S10**
3. **Catalyst characterizations (XRD and XPS)……………………………………………S11-S12**
4. **TOF values of catalyst……………………………………………………………………S12**
5. **Faradaic efficiency of bi-CoPc/carbon…………………………………………………S13**
6. **Chronopotentiometry curves of mono-CoPc/carbon…………………………………S13**
7. **Characterization of catalysts before and after reaction……………………………S14-S15**
8. **Photocatalytic OER performance…………………………………………………………S16**
9. **Electrokinetic study for mono-CoPc/carbon……………………………………………S17**
10. **UV-vis spectroelectrochemistry curves of catalysts without applied potential…………..S18**
11. **The free energy for all intermediate species with catalysts……………………………S19-S20**
12. **Attached Tables……………………………………………………………………………S21-S22**
13. **References…………………………………………………………………………………S23**

Experimental

1. Chemical and material

Carbon support (carbon black, Vulcan XC-72) was purchased from Sigma-Aldrich (USA). Potassium hydroxide (KOH), ethanol, acetone, dimethyl formamide (DMF), acetonitrile (CN_3CN) were purchased from Sinopharm Chemical Reagent Co. Ltd. (Shanghai, China). The mononuclear cobalt phthalocyanine (mono-CoPc) and binuclear cobalt phthalocyanine (bi-CoPc) were purchased from D-chem Ltd (Guangzhou, China). The water used in all experiments was ultrapure (18.2 MΩ). The Ar (99.99%) were purchased from Guangming Gas (Dalian, China). Graphite plate was purchased from Haimen shuguang carbon industry Co. Ltd (Haimen, China).

2. Synthesis of mono-CoPc/carbon and bi-CoPc/carbon

Synthesis of bi-CoPc/carbon

The bi-CoPc/carbon was prepared by mixing the carbon and bi-CoPc in dimethyl formamide (DMF). In a typical procedure, 2 mg bi-CoPc was dissolved in 20 ml of DMF, and the solution was added into the bottle including 50 mg carbon black powder. The mixture was stirred for 48 hours at room temperature. Then the decolorization of the solution indicated that the complexes were attached onto carbon support. The slurry liquid was centrifuged and washed with ethanol, and dried in a vacuum drying oven at room temperature.

Synthesis of mono-CoPc/carbon

The synthetic process is almost the same with bi-CoPc/carbon. In short, 2.14 mg mono-CoPc was dissolved in 20 ml of DMF, and the solution was added into the bottle including 50 mg carbon black powder. The mixture was stirred for 48 hours at room

temperature. Then the slurry liquid was centrifuged and washed with ethanol, and dried in a vacuum drying oven at room temperature.

3. Characterization

3.1 Characterization of composites

X-ray powder diffraction (XRD) patterns of as-prepared samples were characterized on Rigaku D/Max-2500/PC. The structure of catalysts structure were determined by using Cu-Kα radiation with a working voltage of 40 kV and current of 200 mA. The patterns were recorded in the range of 20-80° with a scan rate of 5°/min. IR absorption spectra were recorded on a Nicolet 470 FT-IR spectrometer equipped with a MCT (HgCdTe) detector. Transmission electron microscope (TEM) images were collected in Hitachi HT7700. High-resolution scanning electron microscopy (HRSEM) was performed in Hitachi S5500. The real loading amount of cobalt in the catalysts was verified by inductively coupled plasma atomic emission spectrometer (ICP-AES) on a Shimadzu ICPS-8100. Before the ICP-AES measurement, 30 mg acid-washed sample was placed in a 50 ml quartz beaker and calcined in an oven at 850 ºC for 2 h to ensure the removal of carbon completely. The residual black matter was dissolved in aqua regia and diluted with water to determine the cobalt loading. The XPS data were collected by a Thermofisher ESCALAB 250Xi instrument with a monochromated Al Kα source (15 kV, 10.8 mA) as X-ray source. Region scans were collected using a 20 eV pass energy (Resolution, FWHM (Ag3d5/2) 0.61eV). And the peak position of C 1s at 284.6 eV was selected as an internal reference. The extended X-ray absorption fine structure (EXAFS) measurements were carried out at 21A X-ray nanodiffraction beamline of Taiwan Photon Source (TPS), National Synchrotron Radiation Research

Center (NSRRC). This beamline adopted 4-bounce channel-cut Si (111) monochromator for mono-beam X-ray nanodiffraction and X-ray absorption spectroscopy. The end-station equipped with three ionization chambers and Lytle/SDD detector after the focusing position of KB mirror for transmission and fluorescence mode X-ray absorption spectroscopy. The photon flux on the sample is range from 1×10^{11}~3×10^{9} photon/sec for X-ray energy from 6-27 keV.

3.2 Electrode preparation

Firstly, 4 mg of sample and 80 mL of 5 wt % Nafion solution were dispersed in 1 mL of 4:1 v/v water/ethanol by sonication 60 min to form a homogeneous solution. Then, 5 μL of the solution was loaded onto the GC electrode of 3 mm in diameter. The final loading for all catalysts on the GC electrodes was about 0.262 mg/cm^2.

We also evaluated the electrocatalytic water oxidation activities of catalysts with different loading amount of molecules. And the electrode preparation process is as follow: The mono-CoPc and bi-CoPc molecules (2.14 and 2 mg) were separately dissolved in 2 mL DMF to form molecular solutions by sonication for 30 min. Different volumes of molecular solution (250, 500, 750 and 1000 μL) were mixed with 5 mg carbon support, and then diluted with mixture solution (ethyl alcohol: 5 wt% Nafion solution = 50:1) to a total volume of 2 mL, followed by sonication for 30 min. Finally, 5 μL of the catalyst ink was drop-dried onto GC electrode of 3 mm in diameter.

3.3 Electrochemical measurements

All the electrochemical tests were performed in a conventional three-electrode system at an electrochemical station (CHI 440C) with Mercury-mercury oxide electrode (Hg/HgO, 1M KOH solution) as the reference electrode, graphitic plate as

the counter electrode, and bi-CoPc/carbon or mono-CoPc/carbon on glassy carbon (GC) electrode as the working electrode. Linear sweep voltammetry with a scan rate of 5 mV/s was conducted in 1 M KOH. All potentials measured were calibrated to RHE using the following equation: E (vs RHE) = E (vs Hg/HgO) + 0.098V +0.0591 pH. Chronopotentiometry measurements (j = 10 mA/cm^2) were performed to evaluate the long-term stability of the materials, the work electrode was prepared by spraying the catalyst ink onto a carbon sheet with a areas of 1×1 cm^2.

3.4 Electrochemical impedance spectra measurements

From the potential range of water oxidation, we chose a applied potential window ranging from 1.4 to 1.6 V vs RHE. A single parallel resistor-capacitor (RC) equivalent circuit shown in Scheme S1 was used for fitting the EIS data (frequency 10^5 -10^0 Hz).

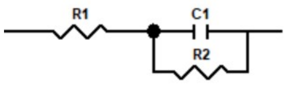

Scheme S1. The equivalent circuit for fitting the EIS data.

3.5 Faradaic efficiency measurements

The Faradaic efficiency test of bi-CoPc/carbon catalyst was measured in a closed pyrex glass reactor at a constant anodic current density of 10 mA/cm^2. Continuous gas flow into the whole reaction system was maintained by using a constant Ar airflow. Quantitative analysis of produced O_2 was online measured by gas chromatography (Agilent 7890 A) using a thermal conductivity detector. The Faradaic efficiency of the OER was calculated as the ratio of the amount of experimentally collected gas to the theoretically generated gas expected from the charge transfer.

3.6 Turnover frequency (TOF) calculation based on electroactive catalyst

In order to calculate the TOF of catalysts for OER, the amount of Co in the catalyst was obtained by ICP and the amount of oxygen was obtained in term of the catalytic current and faradaic efficiency for OER, here the faradaic efficiency is 100%.

$$TOF = \frac{d(n_{O_2})}{Ndt} \qquad (1)$$

where N (mol) is the amount of active sites, here is the Co atoms. n_{O_2} refers to the amount of oxygen.

Considering that the generation of one equivalent of oxygen requires 4 electron transfer:

$$n(O_2) = \frac{1}{4}n(e^-) \qquad (2)$$

therefore,

$$TOF = \frac{d(n_{O_2})}{Ndt} = \frac{d(n_{e^-})}{4Ndt} \qquad (3)$$

According to:

$$Q = It = F * n(e^-) \qquad (4)$$

F is Faraday's constant (96485 C/mol), $n(e^-)$ is the amount of electron and I refers to the catalytic water oxidation current.

then:

$$TOF = \frac{d(n_{e^-})}{4Ndt} = \frac{I}{4NF} \qquad (5)$$

3.7 UV−vis spectroelectrochemistry technique

The UV−vis spectroelectrochemistry measurement of catalyst was performed in a polytetrafluoroethylene reactor. The electrochemical tests were performed in a

conventional three-electrode system at an electrochemical station (CHI 440C) with Silver-silver chloride electrode (Ag/AgCl, Saturated potassium chloride solution) as the reference electrode, platinum wire as the counter electrode, and bi-CoPc or mono-CoPc on fluorine-doped tin oxide (FTO) electrode as the working electrode. Firstly, 2 mg of bi-CoPc (mono-CoPc: 2.14mg) dispersed in 5 mL of DMF by sonication 20 min. Then, the work electrode was prepared by spreading the 50 μL catalyst solution onto the FTO electrode of 1×4 cm^2. Linear sweep voltammetry with a scan rate of 5 mV/s was conducted in 1 M KOH . All potentials measured were calibrated to RHE using the following equation: E (vs RHE) = E (vs Ag/AgCl) + 0.197V +0.0591pH.

UV–vis diffuse reflection spectra of the samples were obtained using a UV–vis spectrophotometer (JASCO V-650) in the wavelength range of 350–900 nm.

4. Computational details for calculations

All of the calculations were performed on the homemade Cluster at DICP using the Gaussian 16 software package. The geometries were optimized with the spin-unrestricted formalism using the B3LYP functional[1] with DFT-D3 (BJ) corrections[2] and 6-311G* basis set[3, 4] for all atoms. Frequency calculations were performed on each optimized structure using the same basis set to ensure that it was a minimum on the potential energy surface. Water is used as the solvent, and the SCRF calculations are carried out to simulate the solvent effects through the SMD method.[5]

The energy of the molecules in the solvation was calculated as the total energy plus the energy of compressing an ideal gas from 24.5 L to 1.0 L, which is RT ln(24.5) or 1.9 kcal mol^{-1}.[6-8] For a proton-coupled electron transfer (PCET) process,

$$BH(aq) \rightarrow B(aq) + H^+(aq) + e^-(g)$$

The standard free energy of a proton in solution (G_{sol}^{H+}) is needed. Here, a value of -272.2 kcal mol^{-1} was used, by adding the free energy of solvation of a proton (-265.9 kcal mol^{-1})[9] and the gas-phase Gibbs free energy of the proton (-6.3 kcal mol^{-1}).[10] The measured redox potential E under experimental pH conditions is related to standard conditions (pH = 0) using the Nernst equation:

$$E = E_{sol}^{\theta} - \frac{RT}{F} ln(10) \times \frac{n_{H^+}}{n_e} \times pH$$

where n_e and n_{H+} are the number of electrons and protons involved in the redox reaction, respectively. Here, for a proton-coupled electron transfer (PCET) process, $n_e = n_{H+} = 1$.

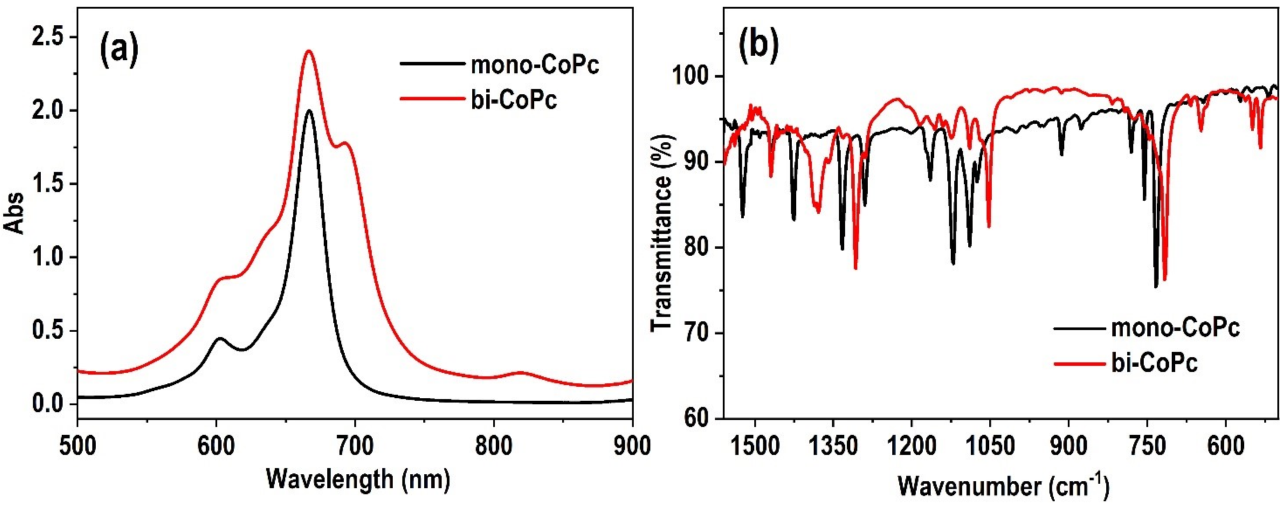

Figure S1. The (a) UV-vis and (b) FT-IR spectra of mono-CoPc and bi-CoPc.

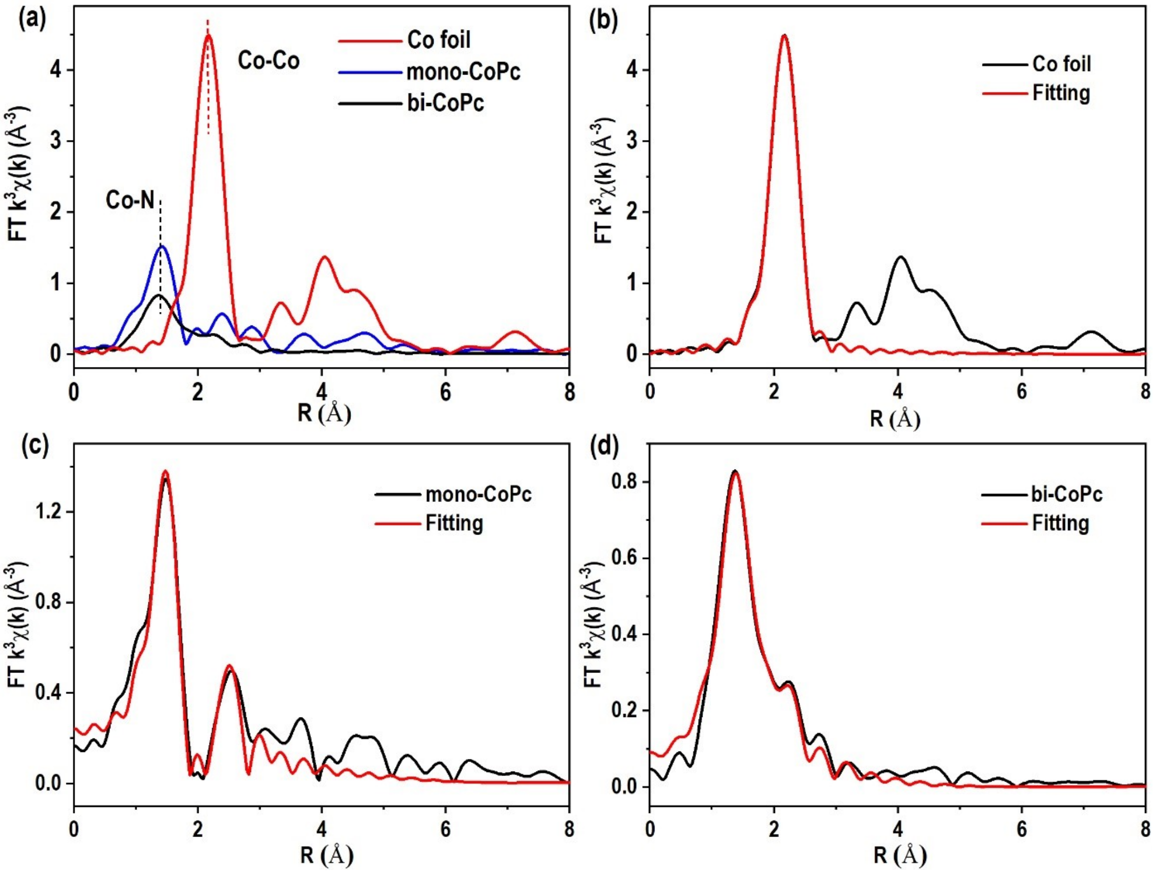

Figure S2. (a) The Fourier transformation (FT)-EXAFS spectra of mono-CoPc and bi-CoPc. The Fourier transform of k^2-weighted EXAFS spectra of (b) Co foil. (c)mono-CoPc and (d) bi-CoPc.

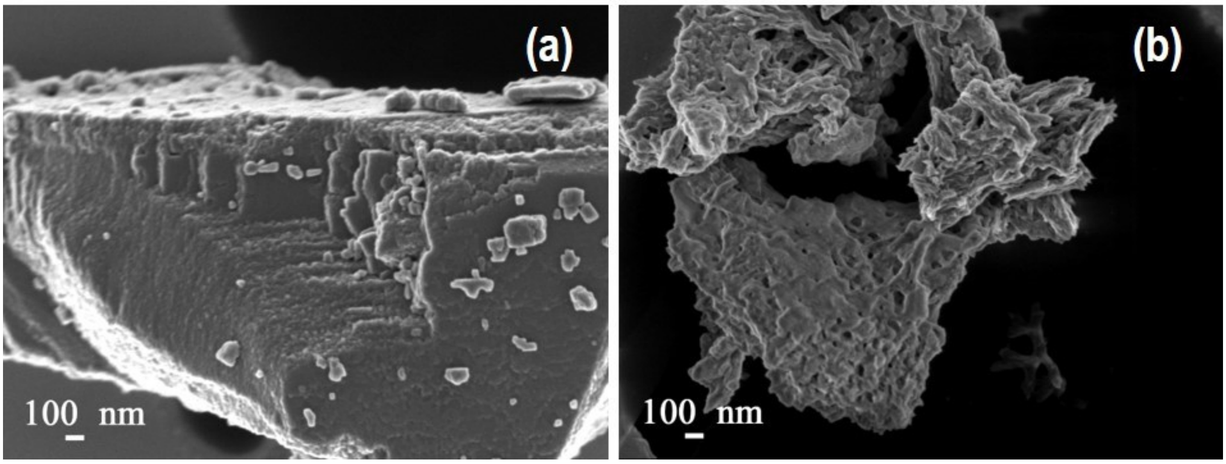

Figure S3. The SEM images of (a) mono-CoPc and (b) bi-CoPc.

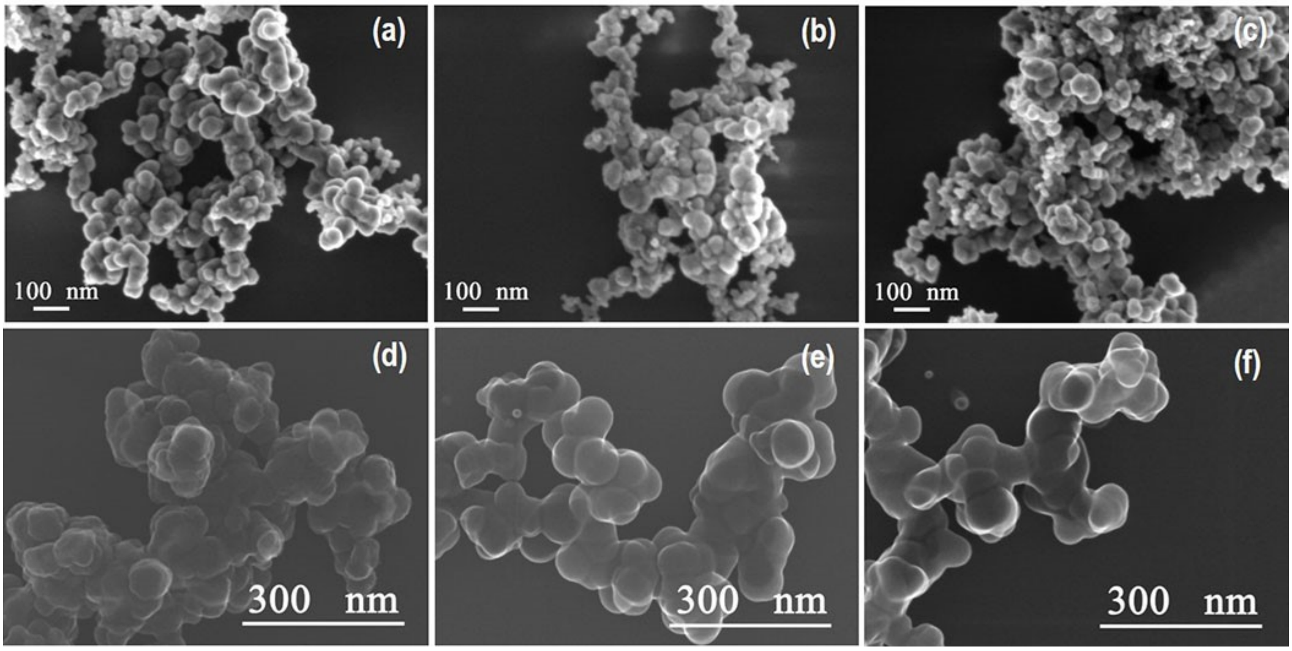

Figure S4. The SEM images of (a) carbon support. (b) mono-CoPc/carbon and (c) bi-CoPc/carbon. The HRSEM images of (d) carbon support. (e) mono-CoPc/carbon and (f) bi-CoPc/carbon

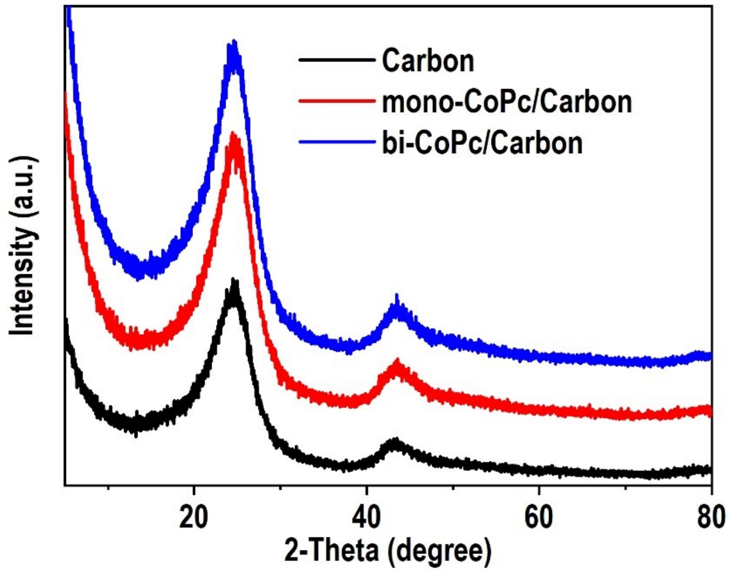

Figure S5. the X-ray diffraction patterns of mono-CoPc/carbon and bi-CoPc/carbon. Reference code for graphite: JCPDS #75-2078.

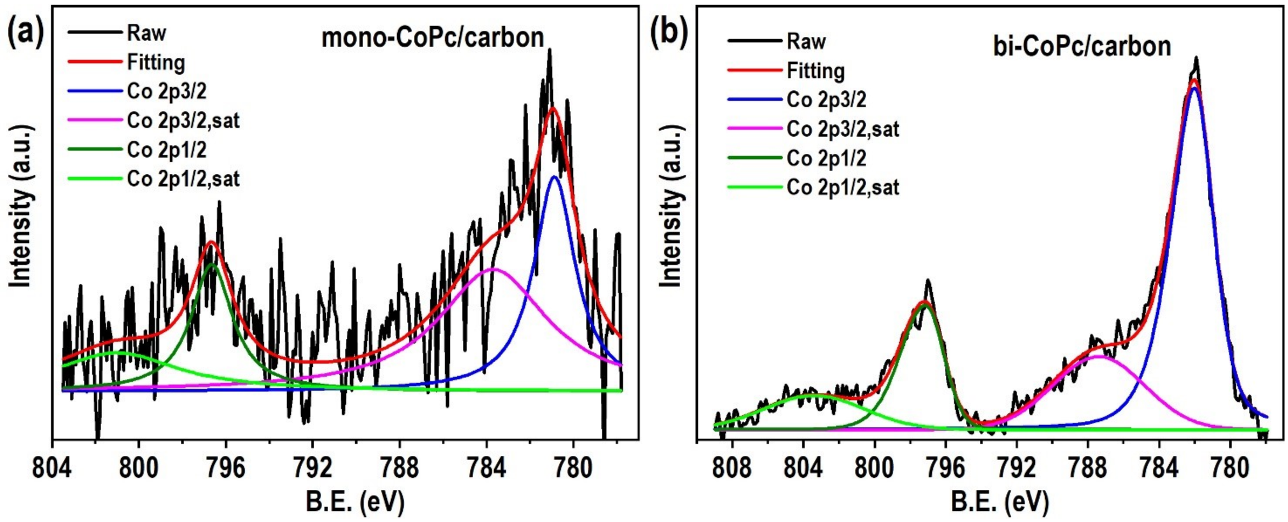

Figure S6. The structural characterizations of catalysts. The XPS spectra of Co 2p for (a) mono-CoPc/carbon and (b) bi-CoPc/carbon.

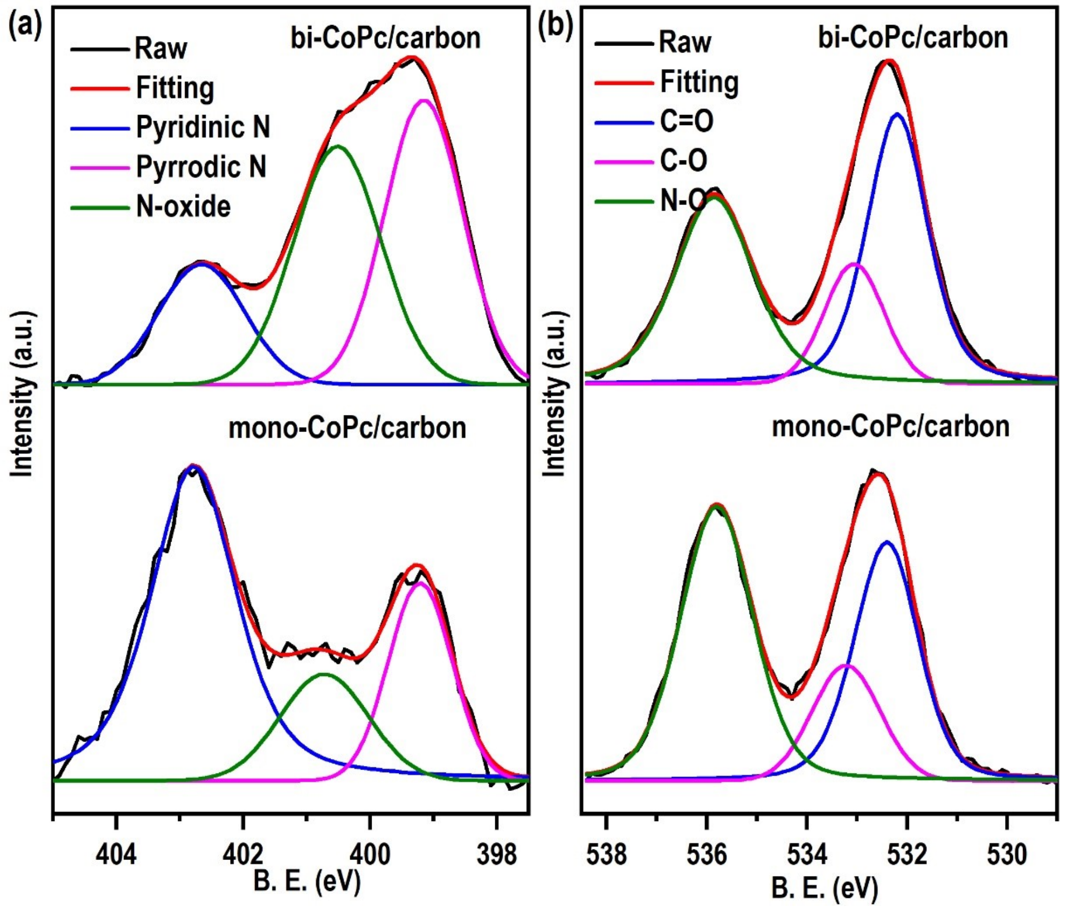

Figure S7. The XPS spectra of mono-CoPc and bi-CoPc. (a) the N 1s spectra of before and reaction. (b) the O 1s spectra of before and reaction.

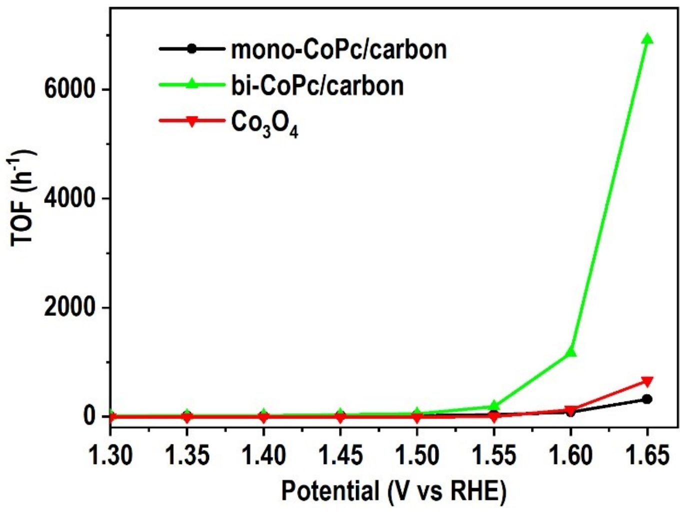

Figure S8. The intrinsic activities of mono-CoPc/carbon, bi-CoPc/carbon and Co_3O_4.

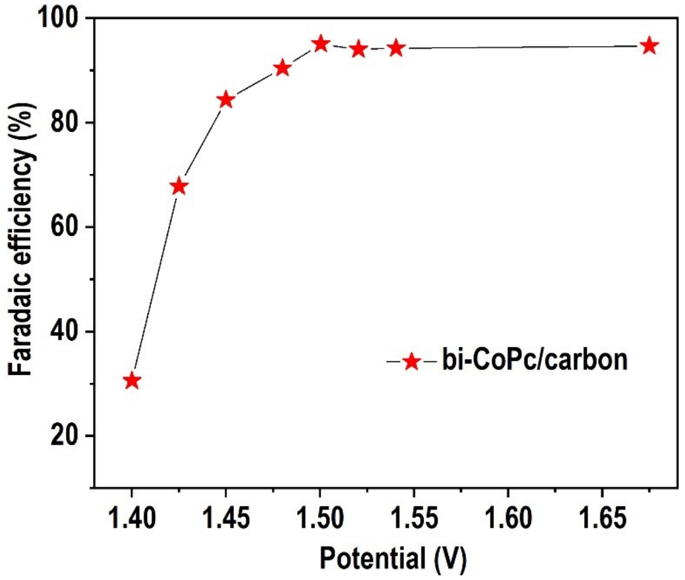

Figure S9. The Faradaic efficiency of bi-CoPc/carbon for water oxidation reaction at different potentials.

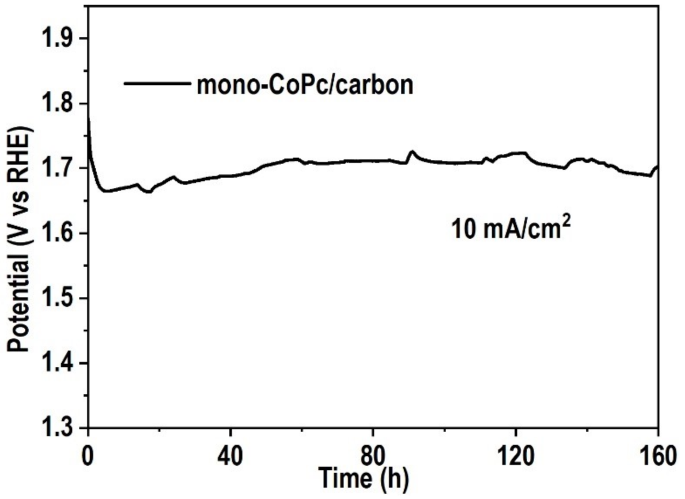

Figure S10. The chronopotentiometry curves of mono-CoPc/carbon at an anodic current density of 10 mA/cm^2.

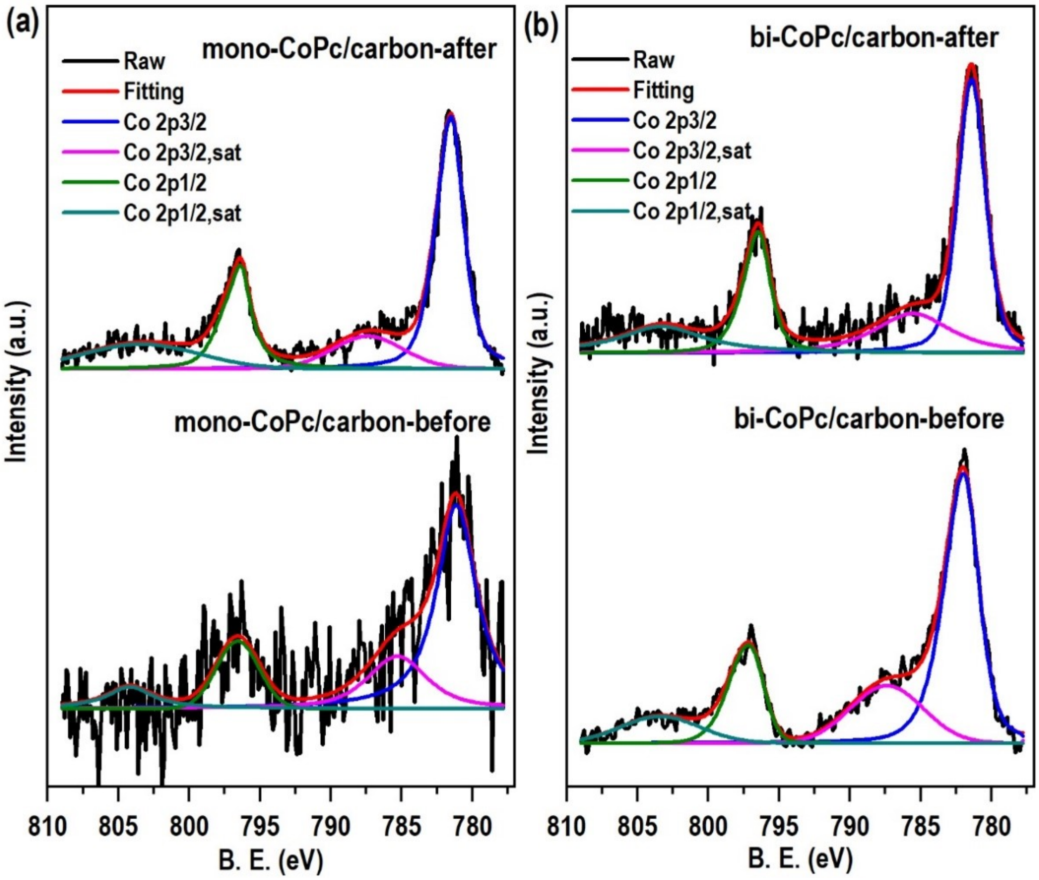

Figure S11. The XPS spectra of mono-CoPc/carbon and bi-CoPc/carbon. (a) the Co 2p spectra of mono-CoPc/carbon before and after reaction. (b) the Co2p spectra of bi-CoPc/carbon before and after reaction.

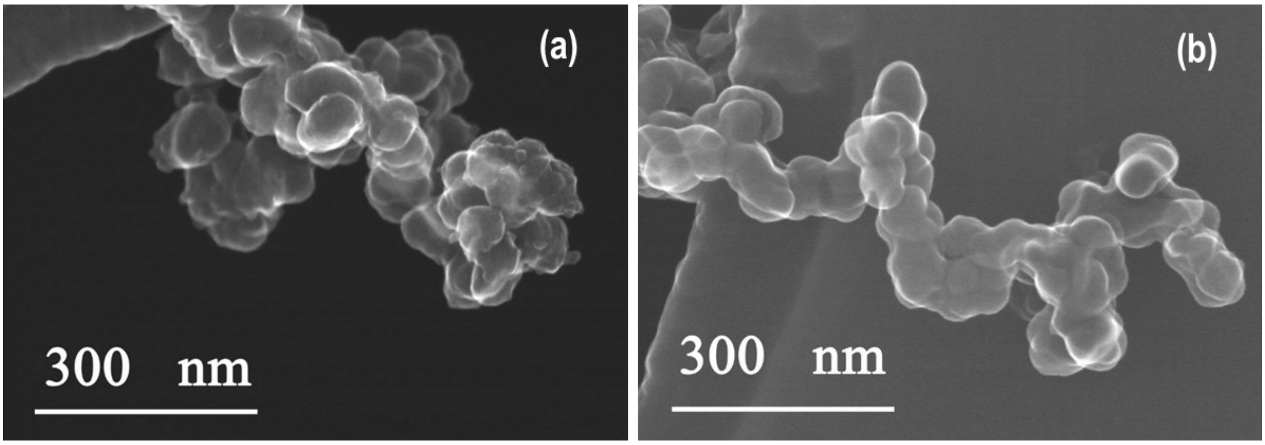

Figure S12. The HRSEM images of bi-CoPc/carbon electrode (a) before and (b) after OER measurement.

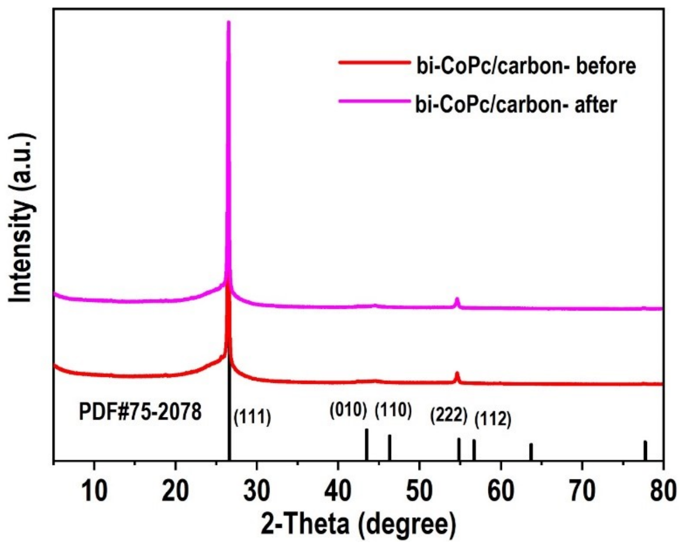

Figure S13. The XRD patterns of bi-CoPc/carbon electrode before and after OER measurement.

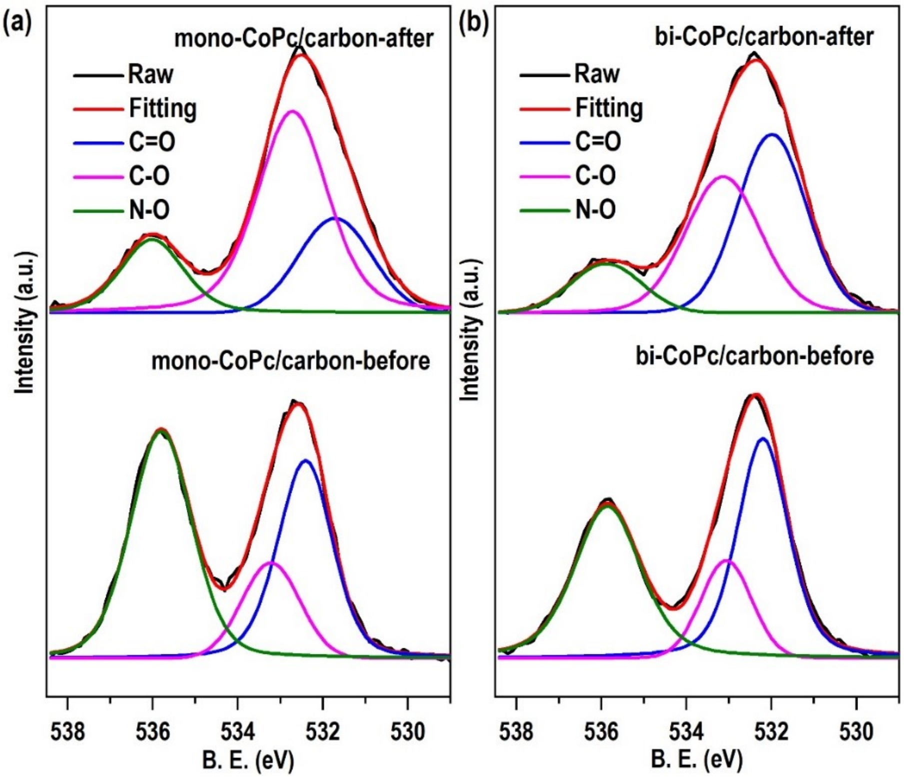

Figure S14. The XPS spectra of mono-CoPc/carbon and bi-CoPc/carbon. (a) the O 1s spectra of mono-CoPc/carbon before and after reaction. (b) the O 1s spectra of bi-CoPc/carbon before and after reaction.

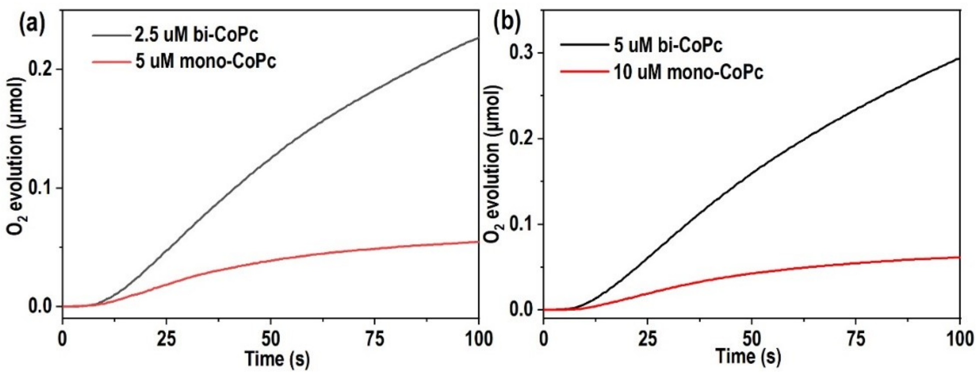

Figure S15. Photocatalytic water oxidation activities of mono-CoPc and bi-CoPc with (a) 5 uM Co sites and（b）10 uM Co sites in the reactor using $Ru(bpy)_3^{2+}$- $Na_2S_2O_8$ system. All photocatalytic activity tests were performed in 50 mM borate buffer (pH 8.0, 3 mL) containing 0.2 mM $Ru(bpy)_3^{2+}$ and 7 mM $Na_2S_2O_8$, and the mono-CoPc or bi-CoPc are directly used as catalysts. For comparison, the same amount of metal sites are used when compared with the catalytic activities of mono-CoPc and bi-CoPc.

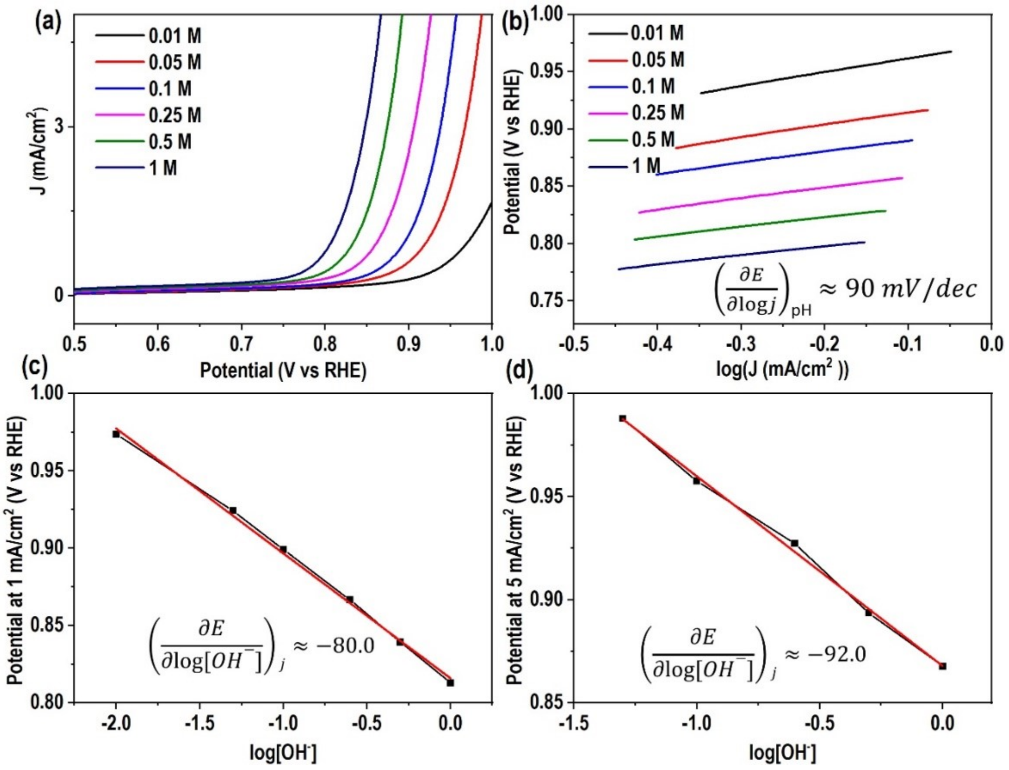

Fig S16. Electrokinetic study for mono-CoPc/carbon. (a) Electrochemical activities in KOH solutions of various concentrations (1.00, 0.50, 0.25, 0.10, 0.05, 0.01 M) with mono-CoPc/carbon. (b) Tafel plots at various pHs. (c) Fitting plot of the potentials at 1 mA/cm^2 versus the logarithm of [OH$^-$]. (d) Fitting plot of the potentials at 5 mA/cm^2 versus the logarithm of [OH$^-$]

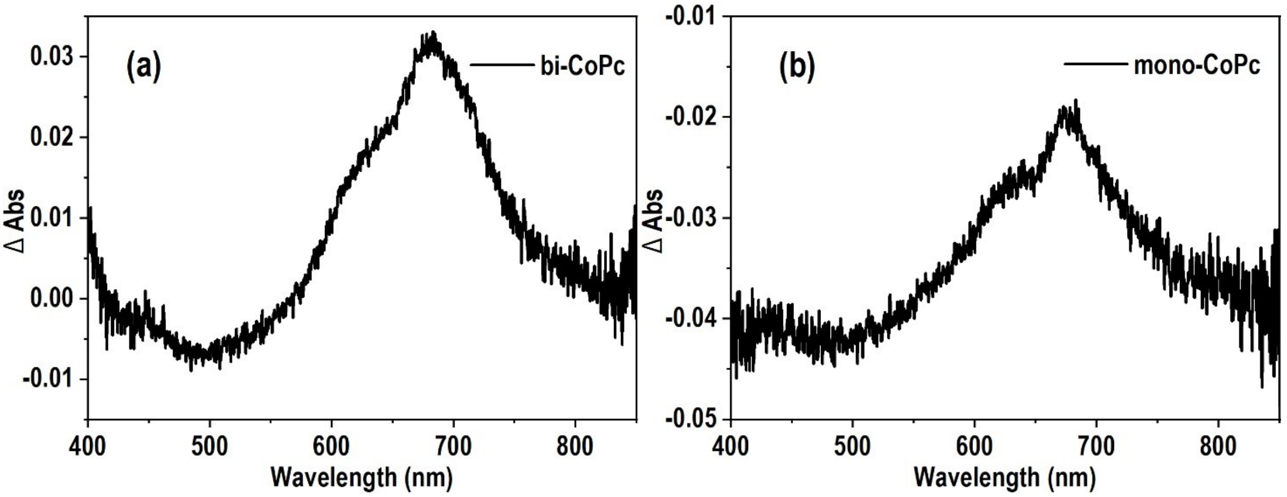

Figure S17. The UV–Vis spectroelectrochemistry spectra of (a) bi-CoPc and (b) mono-CoPc without applied potential.

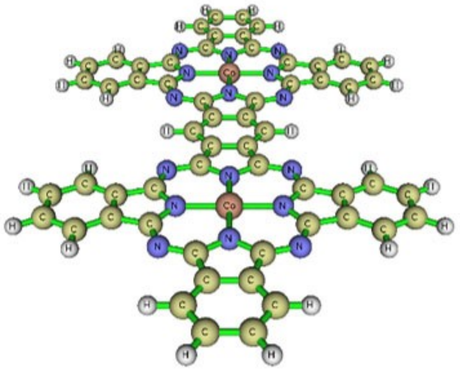

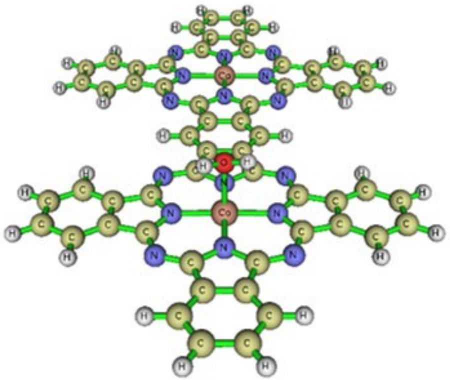

a: Co^{II}-Pc-Pc-Co^{II} (-159679.628) b: Co^{II}-Pc-Pc-Co^{II}-OH_2 (-161760.364)

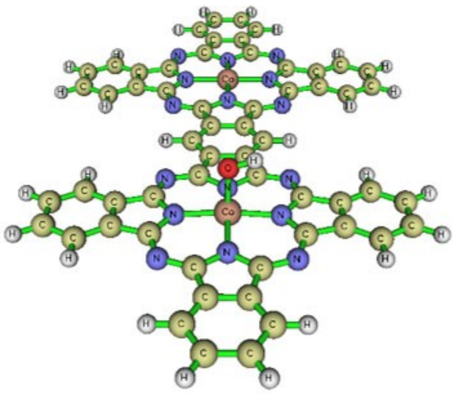

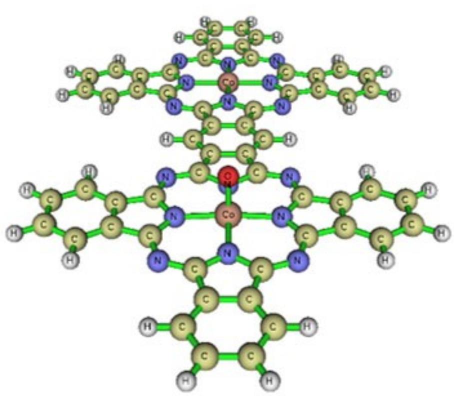

c: Co^{II}-Pc-$Pc^{\cdot +}$-Co^{II}-OH (-161742.802) d: Co^{II}-Pc-$Pc^{\cdot +}$-Co^{II}-O· (-161725.049)

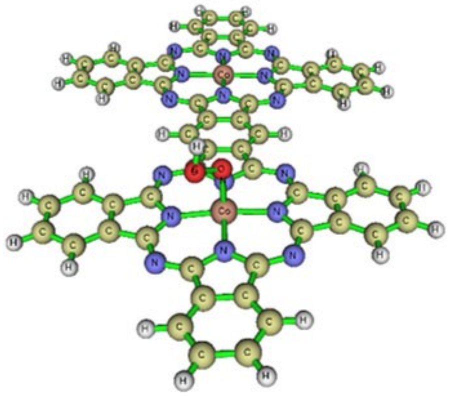

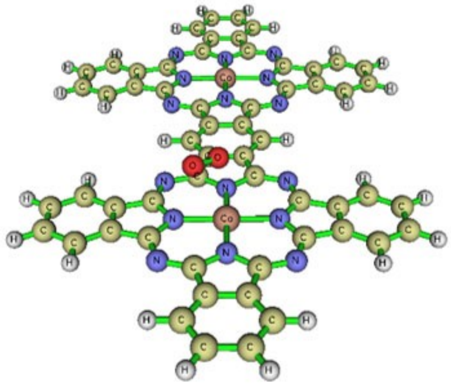

e: Co^{II}-Pc-$Pc^{\cdot +}$-Co^{II}-OOH (-163788.109) f: Co^{II}-Pc-Pc-Co^{II}-OO· (-163771.821)

Figure S18. Computed geometries and free energies (eV) of the water oxidation reaction intermediate species for bi-CoPc.

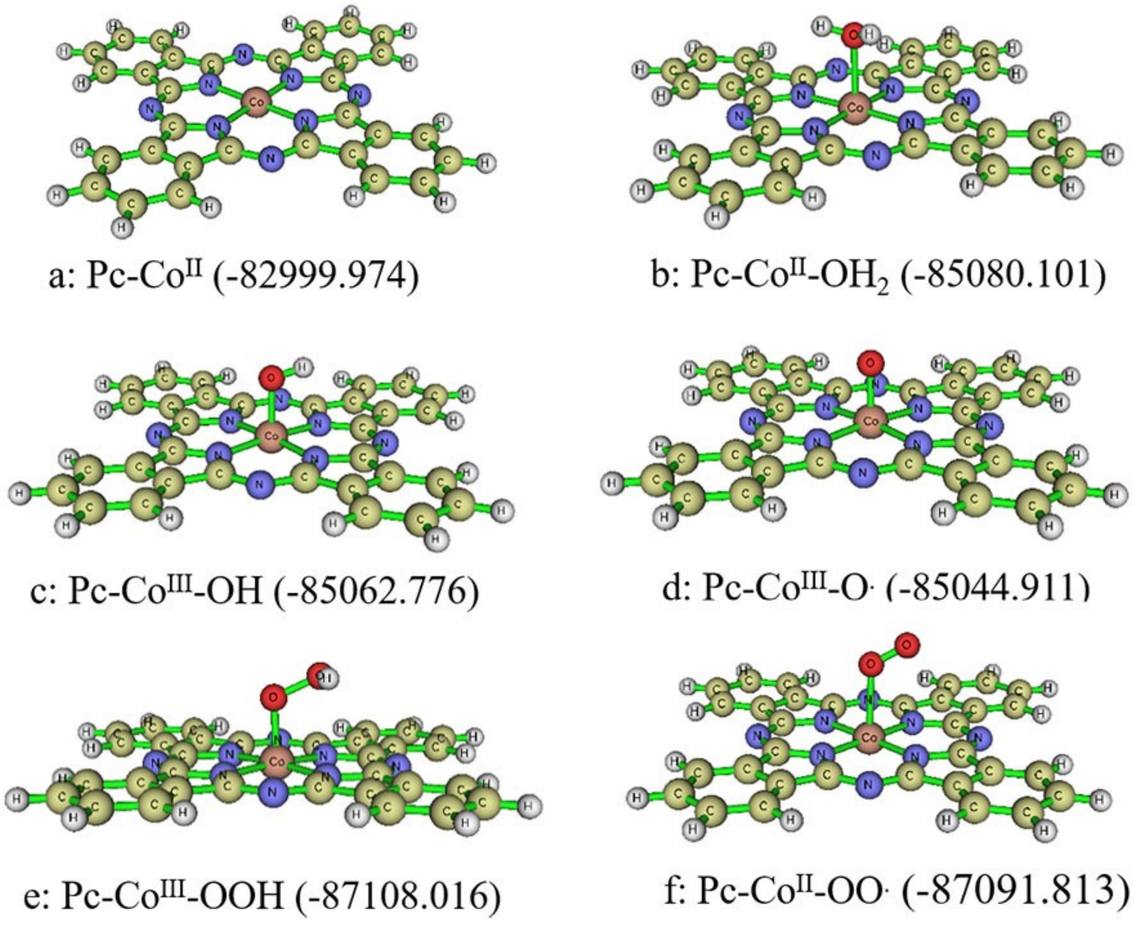

Figure S19. Computed geometries and free energies (eV) of the water oxidation reaction intermediate species for mono-CoPc.

Table S1. EXAFS data fitting results of the Co foil, mono-CoPc and bi-CoPc

	shell	CN	R(Å)	σ^2	ΔE_0	R factor
Co foil	Co-Co	12	2.49±0.01	0.0064	7.5±0.4	0.0023
mono-CoPc	Co-N	4.2±0.2	1.91±0.01	0.0015	3.2±1.9	0.0160
	Co-C	8.4±1.1	2.95±0.01	0.0037		
bi-CoPc	Co-N	4.1±0.2	1.93±0.01	0.0105	-3.6±1.2	0.0059
	Co-C	5.6±0.9	2.74±0.02	0.0080		

^aN: coordination numbers; ^bR: bond distance; $^c\sigma^2$: Debye-Waller factors; ^{d} ΔE_0: the inner potential correction. *R* factor: goodness of fit.

The obtained XAFS data was processed in Athena (version 0.9.26) for background, pre-edge line and post-edge line calibrations. Then Fourier transformed fitting was carried out in Artemis (version 0.9.26). The k^3 weighting, k-range of 3 - 14 $Å^{-1}$ and R range of 1 - ~3 Å were used for the fitting of Co foil; k-range of 3 - 12 $Å^{-1}$ and R range of 1 - ~3.5 Å were used for the fitting of samples. The four parameters, coordination number, bond length, Debye-Waller factor and E_0 shift (CN, R, ΔE_0) were fitted without anyone was fixed, the σ^2 was set.

Table S2: The pH values of the corresponding concentration of KOH

The concentration of KOH (M)	pH values
1.00	13.9
0.50	13.7
0.25	13.4
0.10	13.1
0.05	12.8
0.01	12.1

Table S3. The free energy for O*, OH* and OOH* adsorbing state on bi-CoPc through metal or phthalocyanine ring oxidized pathway

Catalysts	Free energy (eV)	Free energy (eV)
bi-CoPc (*)	-159679.628	-159679.628
bi-CoPc-H_2O (H_2O*)	-161760.364	-161760.364
bi-CoPc-OH (HO*)	-161742.718	-161742.802
bi-CoPc-O (O*)	-161725.061	-161725.049
bi-CoPc-OOH (HOO*)	-163787.945	-163788.109
bi-CoPc-OO (OO*)	-163771.821	-163771.821
	**Metal oxidized pathway**	**Ring oxidized pathway**

The phthalocyanine ring oxidized pathway is favored for water oxidation on bi-CoPc.

Table S4. The free energy for O*, OH* and OOH* adsorbing state on mono-CoPc through metal or phthalocyanine ring oxidized pathway

Catalysts	Free energy (eV)	Free energy (eV)
mono-CoPc (*)	-82999.974	-82999.974
Mono-CoPc-H_2O (H_2O*)	-85080.101	-85080.101
mono-CoPc-OH (HO*)	-85062.776	-85062.559
mono-CoPc-O (O*)	-85044.911	-85044.800
mono-CoPc-OOH (HOO*)	-87108.016	-87107.903
mono-CoPc-OO (OO*)	-87091.813	-87091.813
	**Metal oxidized pathway**	**Ring oxidized pathway**

The metal oxidized pathway is favored for water oxidation on mono-CoPc.

1 A. D. Becke, *J. Chem. Phys.*, 1993, **98**, 1372-1377.
2 S. Grimme, J. Antony, S. Ehrlich and H. Krieg, *J. Chem. Phys.*, 2010, **132**, 154104.
3 K. Raghavachari and G. W. Trucks, *J. Chem. Phys.*, 1989, **91**, 1062-1065.
4 R. Krishnan, M. Frisch and J. Pople, *J. Chem. Phys.*, 1980, **72**, 4244-4245.
5 A. V. Marenich, C. J. Cramer and D. G. Truhlar, *J. Phys. Chem. B*, 2009, **113**, 6378-6396.
6 M. Z. Ertem and C. J. Cramer, *Dalton Trans.*, 2012, **41**, 12213-12219.
7 M. Z. Ertem, L. Gagliardi and C. J. Cramer, *Chem. Sci.*, 2012, **3**, 1293-1299.
8 L. Vigara, M. Z. Ertem, N. Planas, F. Bozoglian, N. Leidel, H. Dau, M. Haumann, L. Gagliardi, C. J. Cramer and A. Llobet, *Chem. Sci.*, 2012, **3**, 2576-2586.
9 M. D. Tissandier, K. A. Cowen, W. Y. Feng, E. Gundlach, M. H. Cohen, A. D. Earhart, J. V. Coe and T. R. Tuttle, *J. Phys. Chem. A*, 1998, **102**, 7787-7794.
10 L.-P. Wang, Q. Wu and T. Van Voorhis, *Inorg. Chem.*, 2010, **49**, 4543-4553.
